# The Importance of Awareness, Acceptance, and Alignment With the Self: A Framework for Understanding Self-Connection

**DOI:** 10.5964/ejop.3707

**Published:** 2022-02-25

**Authors:** Kristine Klussman, Nicola Curtin, Julia Langer, Austin Lee Nichols

**Affiliations:** 1 Connection Lab, San Francisco, CA, USA; University of South Wales, Treforest, Wales, United Kingdom

**Keywords:** self-connection, connection, theory, well-being, mental health

## Abstract

We provide a theoretical framework for what it means to be self-connected and propose that self-connection is an important potential contributor to a person’s well-being. We define self-connection as consisting of three components: 1) an awareness of oneself, 2) an acceptance of oneself based on this awareness, and 3) an alignment of one’s behavior with this awareness. First, we position the concept within the broader self literature and provide the empirical context for our proposed definition of self-connection. We next compare and contrast self-connection to related constructs, including mindfulness and authenticity. Following, we discuss some of the potential relationships between self-connection and various aspects of mental health and well-being. Finally, we provide initial recommendations for future research, including potential ways to promote self-connection. In all, we present this theory to provide researchers with a framework for understanding self-connection so that they can utilize this concept to better support the efforts of researchers and practitioners alike to increase individuals’ well-being in various contexts.

In recent years, there has been growing interest in understanding the factors that contribute to people experiencing meaningful and happy lives (e.g., Lyubomirsky, Sheldon, & Schkade, 2005; Seligman, 2002, 2008; Veenhoven, 2003). Results suggest that a fulfilling life includes a sense of meaning (Steger, 2009), strong interpersonal relationships (Myers, 2000), and the pursuit and attainment of personal goals (Emmons, 2003). In addition, there is a long-standing belief that happiness is the result of identifying one’s strengths and virtues and living a life that cultivates and reflects them (Aristotle, 2002).

In our research lab, we consider how people build lives characterized by deep connection to self and others and the importance of these efforts for health and well-being. People often understand, and empirical research supports, the importance of establishing close relationships and building social networks (Cohen, 2004; Helliwell & Putnam, 2004; Holt-Lunstad & Smith, 2012; Lakey & Cronin, 2008; Lakey, Vander Molen, Fles, & Andrews, 2016). In contrast, although “knowing oneself” has long been of philosophical and psychological interest, sparse research has investigated what self-connection is and what it means to people’s health and well-being. In this paper, we will 1) provide an overview of the definition of self-connection and its three components, 2) discuss the potential well-being increases that result from self-connection, and 3) present some initial thoughts on the fruitful directions that future investigations of self-connection might pursue.

## Understanding the Self in Self-Connection

The first, and possibly most important, aspect of self-connection is that it refers to the self. As such, it is useful to clarify our intended use and context of the term “self.” Social psychological theories generally highlight that people form and maintain self-concepts (i.e., ideas about who they are as distinct entities). Self-concepts help individuals to organize information extracted from momentary experiences. Select information is attended to as self-relevant, often information related to autobiographical memories and motivations, and is mentally processed in ways that can yield a sense of more enduring (though still malleable) personal characteristics and social roles (Markus, 1977; Oyserman, 2001). For example, if one currently feels compelled to comfort a distressed acquaintance and recalls multiple instances of feeling concerned for friends, the person’s self-concept might include “caring” or “a supportive friend.” Of note, one may hold multiple overlapping self-concepts (e.g., public and private selves; see Baumeister, 2012) and multi-faceted self-concepts (e.g., situationally contingent or flexible aspects of the self; see Paulhus & Martin, 1988). Within these self-concepts, people often have a sense of what they regard as their true self, in terms of what is most essential about them or most personally endorsed (Rogers, 1959; Schlegel & Hicks, 2011). This perceived true self is important for our definition of self-connection.

Both the perceived true self and potentially broader self-concepts are formed and maintained by selectively attending to, interpreting, and remembering aspects of momentary experiences (see Oyserman, 2001). In turn, maintaining one’s self-concept can sometimes detract from individual and social well-being, such as when individuals become preoccupied in primarily negative self-related thoughts (Lyubomirsky & Nolen-Hoeksema, 1993; Nolen-Hoeksema, 2000) or react defensively toward others (Crocker & Park, 2004; Greenberg, Solomon, & Pyszczynski, 1997). To avoid such undesirable efforts of self-concept maintenance, some research has explored meta-cognitively distancing (e.g., “disidentifying”) from self-related thinking during momentary experiences (Bernstein et al., 2015; Fresco et al., 2007; also see Brewer, Garrison, & Whitfield-Gabrieli, 2013). Although these approaches may be useful in some instances, they risk throwing out the baby with the bathwater, so to speak, since engaging with the self can be beneficial when it helps people find meaning and purpose in life (Schlegel & Hicks, 2011; Schlegel, Hicks, King, & Arndt, 2011). This then raises the question of if that upside can be maximized while minimizing potential pitfalls. Within that context, this paper presents the concept of self-connection as a way of relating to the self that supports individual and social well-being.

## Defining Self-Connection

In addition to its focus on the self, self-connection inherently involves a sense of connection. In our conceptualization, that sense of connection uniquely relates to the perceived self. More specifically, it refers to the presence of and relationship between three capacities—awareness of, acceptance of, and behavioral alignment with oneself. Consequently, we define self-connection as a subjective experience consisting of three components: 1) an awareness of oneself, 2) an acceptance of oneself based on this awareness, and 3) an alignment of one’s behaviors with this awareness. We posit that the three components are interrelated in a non-hierarchical structure and contribute synergistically to experiencing self-connection. As such, an individual who is lacking in any of the three components would experience less overall self-connection.

### Awareness of Oneself

The first component of self-connection, self-awareness, is defined as knowing one’s internal states, preference, resources, and intuitions (Goleman, 2006). As part of their self-concepts, many people believe that they have an essential, internal, and private self, capable of being truly or fully known only to them (Rogers, 1959; Schlegel & Hicks, 2011). This perceived self may be more endorsed or important than other aspects of one’s self-concept. People may see this self as immutable, but some psychological perspectives, such as Self-Determination Theory (SDT; Deci & Ryan, 1980, 1985), posit that the self need not be unchangeable but rather be determined and meaningful to the individual (Schlegel & Hicks, 2011). As such, we are not arguing that there is one, “real” internal self (Darley & Fazio, 1980; Murray, Holmes, & Griffen, 1996). Instead, we contend it is people’s perceived understanding of aspects of their self-concepts resembling a self (e.g., important values) that is relevant to experiencing self-connection.

Also informing our conceptualization of the awareness component of self-connection is the construct of mindfulness. One central feature of mindfulness is an awareness of and attention to one’s current experiences, from moment to moment (Bishop et al., 2004; Brown & Ryan, 2003; Kabat-Zinn, 1990). Part of mindfulness is observing or noting sensations, thoughts, and emotions as they occur, bringing them into awareness and potentially greater clarity (Brown, Ryan, & Creswell, 2007; Mikulas, 2011). Similarly, the awareness component of self-connection notices self-relevant aspects of experiences, potentially providing more attention to, and clarity on, those that pertain to oneself.

### Acceptance of Oneself

The second part of the definition of self-connection is self-acceptance and can be defined as a complete acceptance of one’s internal states, preference, resources, and intuitions. Acceptance involves receptivity and openness to oneself, rather than avoidance and denial. We assert that acceptance of the perceived self is a key component of self-connection and can also best be understood within the psychological literature on mindfulness. A second, integral quality of mindfulness, in addition to present-moment awareness, is an accepting stance toward experiences (see Kabat-Zinn, 1990; Lindsay & Creswell, 2017). Acceptance in mindfulness involves receptively meeting one’s experiences as they are, without trying to alter them. Self-connection consists of a similar acceptance, yet this acceptance is oriented more toward the self. When self-relevant experiences and characteristics come into awareness, they are allowed as “this feels like part of me” and not automatically judged as good or bad. In this way, the acceptance component of self-connection is not about liking or esteeming oneself (or how likable the self is to others), as some other conceptualizations of self-acceptance include (e.g., Ryff & Keyes, 1995). Instead, the focus is on a willingness to acknowledge one’s feelings, values, and other aspects of the self and truly accepting oneself.

### Alignment With Oneself

The third part of our definition involves drawing on one’s awareness and acceptance of the perceived self when making behavioral decisions. This self-alignment can be defined as behaving in ways that are consistent with one’s internal states, preference, resources, and intuitions. Specifically, self-connection involves acting in alignment (component three) with one’s awareness of the self (component one) by using one’s acceptance of this awareness (component two) to facilitate behaviors that align with the perceived self. This component of self-connection is similar to conceptualizations of self-determined decisions in Self-Determination Theory (Deci & Ryan, 1980, 1985) as well as authentic behavior (Kernis & Goldman, 2006; Wood, Linley, Maltby, Baliousis, & Joseph, 2008). That is, behavioral alignment involves deciding to act in ways that authentically reflect the perceived self. Awareness and acceptance of the perceived self theoretically should facilitate aligned behavioral decisions, and behavioral experiences may also help individuals to become more aware of what they perceive as their self and/or accept that self. Developing concordance between behavior, self-awareness, and self-acceptance is critical to a lived experience of self-connection.

## Limitations of Similar Concepts

### Self-Connection Versus Authenticity

In part, the proposed definition of self-connection shares some relation with concepts of authenticity (Kernis & Goldman, 2006; Wood et al., 2008) but can also be distinguished from them. In their development of a measure of dispositional authenticity, Wood and colleagues (2008) argued that authenticity primarily includes authentic living—the degree to which a person’s behavior matches their self. This most strongly maps onto the self-alignment component of self-connection, but is only one of three necessary components of self-connection. Other conceptualizations or operationalizations of authenticity also may include some form of awareness or acceptance (see Kernis & Goldman, 2006). These differ from how we conceptualize awareness and acceptance in self-connection. First, self-connection is inherently relational within one’s own experience: It is about experiencing a sense of linkage with oneself. That is, awareness and acceptance are essentially a way of relating to one’s self-relevant mental processing and tuning into oneself. In contrast, conceptualizations of awareness and acceptance in authenticity tend to imply that one exists in an experience of the self and are more focused on avoiding self-deception and contending with external influences and judgments, respectively (Kernis & Goldman, 2006). Authenticity may also be affected by judgments of “good” and “bad” whereas these are not relevant to self-connection. Likewise, we assert that the nonjudgmental conceptualization of acceptance proposed as part of self-connection may have added value in enhancing one's ability to act in alignment with oneself.

### Self-Connection Versus Mindfulness

The proposed definition of self-connection bears resemblance to mindfulness but also contains aspects that distinguish the two concepts. Awareness and a lack of judgement are two defining, synergistic components of mindfulness (see Lindsay & Creswell, 2017). However, most scientific definitions of mindfulness do not include alignment of behavior with the perceived self (the third component of self-connection). When intentional behavior is considered, it is usually as a correlate or consequence of mindfulness (e.g., Chatzisarantis & Hagger, 2007). Moreover, mindfulness itself does not specifically concern or reference the self, as self-connection does. In fact, substantial mindfulness-related theory and research addressing the self treats it as something to distance oneself from or to transcend (see Bernstein et al., 2015). A growing literature does address mindful self-compassion, but this concept only concerns handling difficult experiences and includes identifying less with them (Neff, 2003). Altogether, we propose that mindfulness concerns itself with broader awareness and acceptance of experience and thus may be helpful for, but is not synonymous with, experiencing self-connection. The concept of self-connection goes beyond mindfulness and self-compassion in that it draws on components of mindfulness—awareness and acceptance—along with behavioral alignment to facilitate experiences of connection to the perceived self.

## Is Self-Connection a State or Trait?

At a basic level**,** self-connection could be thought of as both a state and an individual difference characteristic (similar to a trait). That is, it is possible to temporarily experience a state of greater self-connection. Additionally, repeatedly experiencing states of increased self-connection may promote its ease and frequency throughout life. Likewise, individuals may differ in the extent to which they generally tend toward experiencing self-connection. Whether this would be considered a trait-level difference might vary with different models of personality. We discuss one such framework next.

Within the context of McAdams and Pals’ (2006) holistic model of personality, self-connection can be understood as a characteristic adaptation—more individualized and, possibly, more malleable across situations and time than a basic trait. In this model, self-connection may be a third- and/or fourth-level characteristic adaptation. Third-level characteristic adaptations are not simply basic traits and instead include “aspects of human individuality that speak to motivational, social-cognitive, and developmental concerns” (p. 208). Awareness of the perceived true self can be developed, and one may choose to accept it and act accordingly or not — these are individual motivational, social-cognitive, and developmental concerns akin to third-level characteristic adaptations. For example, as one might develop a commitment to environmental conservation (itself a characteristic adaptation), one might simultaneously develop awareness and acceptance of that value and act in a manner consistent with it.

The fourth level of McAdams and Pals’ (2006) model refers to the more malleable aspects of characteristic adaptations that are subject to change based on context or experience. Characteristic adaptations are more likely to change over time than traits as they are anchored in everyday situational and personality processes and dynamics. Awareness of, acceptance of, and alignment with a value may develop nuances as the value is experienced in more contexts—a fourth-level characteristic adaptation. As such, self-connection may contribute in significant ways to an individual's development across life domains. To the extent that people experience self-connection across life domains and throughout daily life, it would be more trait-like for an individual, even though any individual could also experience a temporary, heightened state of self-connection. As such, we view self-connection as something that can be treated and examined at both the state and trait level.

## The Implications of Self-Connection for Well-Being

We propose that self-connection is a way of relating to the self that supports positive functioning and well-being. Specifically, experiencing connection to oneself should promote meaning and purpose in life and greater attainment of related goals. In terms of well-being assessment, this may also be reflected in greater life satisfaction (as in assessments of subjective well-being; Diener, 1984) and greater eudaimonic well-being at a personal level (as operationalized as either flourishing as in Keyes, 2002, or psychological well-being as in Ryff, 1989). Self-connection may also conceivably enhance various aspects of social connection and social well-being. For example, one may be able to communicate preferences and values to others more clearly and support others in doing so. More self-connected individuals may also engage in more meaningful social activities due to acting in alignment with their values. For such reasons, connection with self and others may go hand-in-hand.

Theoretical and empirical literatures on related constructs provide indirect support for these propositions. The mindfulness literature suggests that awareness and acceptance are associated with greater well-being (Lindsay & Creswell, 2017; McNall, Tombari, & Brown, 2019). More relevant to self-awareness specifically, Schlegel and colleagues assert that discovering and expressing the self is crucial to psychological health (Schlegel, Hicks, Arndt, & King, 2009). Their research suggests that the feeling of knowing yourself predicts self-actualization, vitality, self-esteem, active coping, psychological need satisfaction, positive affect, and subjective well-being (Schlegel, Vess, & Arndt, 2012). Schlegel and colleagues also assert that understanding the self allows one to interpret actions that are congruent with the self as valuable (Schlegel & Hicks, 2011) and provide a sense of coherence (Hicks, 2013).

Furthermore, beyond research on mindfulness (which is inherently accepting), some research also suggests that self-acceptance may play a role in well-being. Most relevant to self-connection is research that conceptualizes self-acceptance as unconditional and less evaluative, as compared to positive self-evaluations (e.g., Ryff & Keyes, 1995). Such research has found unique, positive associations between self-acceptance and overall mood as well as eudaimonic well-being (Chamberlain & Haaga, 2001; MacInnes, 2006; Ranzijn & Luszcz, 1999).

Several research programs also provide evidence to support the argument that congruence between one’s implicit and/or internal goals and explicit behaviors is an important cornerstone of well-being (Schultheiss & Brunstein, 1999; Schultheiss, Jones, Davis, & Kley, 2008; Sheldon, 2004, 2014). For example, people who choose goals based on their own internal interests are more likely to achieve those goals (Sheldon & Elliot, 1999; Sheldon & Houser-Marko, 2001) and show increased levels of happiness (Sheldon & Elliot, 1998; Sheldon & Kasser, 1998). Bailis, Fleming, and Segall (2005) surveyed people when they first joined a gym and found that people who had self-concordant goals were more likely to be members of the gym 2 years later, were less likely to compare themselves to others, and were less negatively influenced by social comparisons. In their experimental study, Chatzisarantis, Hagger, and Wang (2010) found that their manipulation of self-concordant goal motivation and implementation intention resulted in the highest level of short-term adherence to taking daily multivitamins. Thus, research suggests pursuing goals that reflect one’s self results in greater long-term commitment to those goals, and possibly even greater satisfaction in the pursuit of them. Altogether, such existing literature suggests that constructs related or similar to the three components of self-connection support well-being. Therefore, it is reasonable to propose that the combination of the three components of self-connection may synergize to support well-being.

There also is some indirect evidence to support the idea that such benefits of self-connection may not carry risks of preoccupation in negative self-related thinking or defensive reactions, both of which may undermine individual and social well-being. For example, research on negative rumination has found that private self-reflection can be distinguished from maladaptive rumination (Trapnell & Campbell, 1999), indicating that self-awareness is not inherently a rumination risk. Self-Affirmation Theory (Aronson, Cohen, & Nail, 1999; Sherman & Cohen, 2006; Steele, 1988) has produced considerable evidence that reminders of broader valued aspects of the self (e.g., writing briefly about a core value after a threat to some other aspect of the self; reminders of other important goals when frustrated about a particular goal), can reduce both negative rumination (Koole, Smeets, van Knippenberg, & Dijksterhuis, 1999) and defensive reactions (Sherman & Cohen, 2006).

These findings are generally consistent with the idea that experiencing a sense of connection to aspects of oneself (e.g., values, important goals) may not carry risks of rumination or defensiveness. Further, the mechanisms underlying such effects are not clear empirically. Self-Affirmation Theory suggests that, after an aspect of the self has been threatened, reminders of other values restore a positive view of the self, reducing a need for rumination or defensiveness. We posit that it is also possible that reminders of values could operate through connecting to oneself with acceptance (rather than needing esteem or liking). This potential role of acceptance is supported by the empirical literature on mindfulness.

Mindfulness inherently involves acceptance and is associated with less maladaptive rumination and defensiveness. Many studies have found that trait mindfulness and mindfulness training are associated with less negative rumination and stress (Gu, Strauss, Bond, & Cavanagh, 2015; Paul, Stanton, Greeson, Smoski, & Wang, 2012; Van der Velden et al., 2015). More mindful individuals also may show fewer defensive reactions to self-related threats. For example, in a series of studies on the role of mindfulness in responses to mortality threats (i.e., making thoughts of death salient, thus threatening people’s sense of self), more mindful individuals were less defensive in their responses. In all, evidence suggests that an accepting awareness may attenuate risks of rumination and defensiveness and thus indirectly supports our contention that self-connection may as well.

## Future Research on Self-Connection

There are several promising directions that research on self-connection might take. We describe only a few of them below.

### Operationalizing Self-Connection

The first requirement for researching self-connection is the development of a validated tool to measure it. Ideally, a measure would be able to assess overall self-connection as well as the individual components of self-connection: self-awareness, self-acceptance, and self-alignment. As conceptualized, self-connection should be measured through a composite of items that measures all three components. Additionally, we need to ensure that the measure is reliable and valid, such as testing whether it relates to relevant variables as predicted and its incremental value beyond existing measures. Development of one such measure is underway in our lab.

The ability to experimentally increase self-connection also is needed to help reach causal conclusions about the effects of self-connection. Experimental manipulations could attempt to temporarily boost a state of self-connection or increase an individual’s overall tendency toward self-connection in daily life. The former might be accomplished with brief, one-time exercises and could potentially reveal immediate, albeit temporary, effects of heightened self-connection on state-dependent measures, including in certain contexts or domains. The latter, increasing individual differences in self-connection, may require more extended intervention, potentially with multiple exercises and/or covering multiple life domains. We describe some potential intervention ideas below.

### Building the Nomological Network of Self-Connection

After we understand how to measure self-connection, research into the nomological network of self-connection needs to examine the ways in which self-connection may or may not relate to various aspects of both well-being and health. Such research could examine cross-sectional and prospective relationships between measured self-connection and meaning in life, aspects of individual and social well-being, and goal persistence and attainment. Intervention studies, especially randomized controlled trials, will provide evidence of directionality and potential causality.

#### Individual Differences Predicting Self-Connection

Correlational and prospective studies also should assess individual differences that may predict the self-awareness, self-acceptance, and/or the self-alignment components of self-connection and the overall representation of self-connection. Trait mindfulness is one variable noted previously. Self-concept clarity also has been associated with mindfulness and may characterize individuals higher in self-connection (Hanley & Garland, 2017). Additionally, consistent with SDT and Sheldon’s (2004) argument that self-determination is vital for achieving an integrated self, Thrash and Elliot (2002) found that people high in self-determination also showed higher levels of congruence between implicitly and explicitly measured motives. Self-determination and implicit-explicit motivational concordance may also relate to self-connection. Other individual differences to examine might include basic personality traits, gender, cultural variables, age, ethnicity, and income.

#### Self-Connection and Meaning in Life

For many people, the search for profound self-understanding and a life built around it is an eternal, imperfect pursuit. As positive psychology has begun to offer many answers to the question of how to best promote and enhance well-being, research has turned to the concept of meaning in life (Martela, Ryan, & Steger, 2018; Schlegel et al., 2011; Steger, 2009). We have posited that self-connection increases of a sense of meaning in life. Knowing about and accepting who one perceives one truly is should theoretically lead to an increase in a sense of coherence across one’s life and allow for actions that are in support of one’s values and goals or purpose. When people act in a way that is in alignment with their values and goals, their sense of significance may also increase. Thus, experimentally increasing self-connection should also increase one's sense of meaning in life, whether at a state or trait-like level.

#### Self-Connection and Broader Well-Being

As detailed above, self-connection may predict individual and social well-being at a dispositional level. Greater coherence, meaning, and social connection from self-connection may also contribute to more positive affect in daily life (see Fredrickson, 2013). These relations could be examined using longitudinal and brief intervention studies (e.g., Goodman, Kashdan, Mallard, & Schumann, 2014). Although such positive functioning is the primary hypothesized outcome of self-connection, greater self-connection may also be associated with fewer depression symptoms (given the roles of anhedonia and hopelessness in depression). Therefore, initial prospective studies should examine a range of potential mental health outcomes. As part of such research on self-connection and well-being, it also would be useful to examine whether high self-connection carries less risk of negative rumination and defensiveness than low self-connection or self-disconnection.

#### Self-Connection and Goal Striving

When people’s perceived selves include goals, self-connection may support self-regulation toward those goals. We propose that awareness and acceptance of such goals may foster greater goal clarity and accessibility, while behavioral alignment may promote follow through on intentions and persistence (cf. Mann, De Ridder, & Fujita, 2013). These hypothesized component processes and the role of overall self-connection in goal striving should be examined in future correlational and, ideally, experimental research. One domain in which the relation between self-connection and goal-related processes could be especially important to examine is health behavior. Not only is engaging in health-promoting behavior important for physical health, it could also be another way that self-connection supports overall well-being.

### Promoting Self-Connection

Once we can measure self-connection and begin to understand how it relates to other constructs and aspects of life, self-connection has the potential to be an extremely useful tool for promoting positive life outcomes. To realize these benefits and study its effects using experimental designs, it will be important to examine how to promote self-connection. It is possible that several existing practices, either in isolation or in combination, may be useful for promoting self-connection by increasing self-awareness, self-acceptance, and/or self-alignment.

For example, we have proposed that mindfulness may facilitate the self-awareness and self-acceptance components of self-connection. Future research should examine whether and when mindfulness practices (formal mindfulness meditation or informal mindfulness in various domains of daily life) can lead to greater self-connection. It also would be interesting to consider the role that self-connection may play in the relationship between mindfulness and aspects of well-being.

Another promising way to promote self-connection may be journaling. Daily journals have been widely used across disciplines (Hülsheger, Alberts, Feinholdt, & Lang, 2013; Hülsheger et al., 2014; Pennebaker, Mehl, & Niederhoffer, 2003; Pennebaker & Seagal, 2003) and can provide people the opportunity to become more aware of their internal thoughts and values. This, in turn, should provide a space to accept them and lead to an understanding of themselves to modify actions as needed. Repeated journaling could focus on different life domains to develop and apply self-connection across daily life. Thus, the act of journaling about self-connection may increase self-connection and the positive outcomes potentially associated with it.

Physical activity also may be a means for promoting self-connection. For one, it may help people tune into their sensations and feelings. When done repeatedly, it also may enhance self-connection through building confidence, independence, and/or positive body image to accept internal values and goals (Kaufman, Glass, & Arnkoff, 2009; Lawlor & Hopker, 2001; Taylor, Sallis, & Needle, 1985). Additionally, physical activity in a non-competitive environment might be especially useful because it may allow people to practice being more accepting of themselves and acting accordingly. Finally, when combined with meditation, the effects of activity could be especially pronounced (Edwards & Loprinzi, 2019).

Interventions aimed at increasing self-connection at more of a trait level, throughout daily life, might benefit from incorporating all the above activities with specific guidance aimed at connecting with oneself. Additional options for practices to increase self-connection are also possible and might even be useful in the workplace (Lomas, Medina, Ivtzan, Rupprecht, & Eiroa-Orosa, 2018). As part of research on promoting self-connection, it also may be important to identify potential barriers (either internal or external) to self-connection and how certain practices or beliefs may help to overcome them. Finally, this all must be done in a way that considers the cultural influences in play (Christopher & Hickinbottom, 2008).

## Conclusion

Being self-connected requires one to be aware of the self, accept that self, and act in alignment with it. We argue that self-connection is important to obtaining greater well-being and believe that there currently is significant indirect evidence to support this claim. We detail our conceptualization of self-connection so that future research can test our propositions more directly. We are optimistic about future research to uncover practices, such as mindfulness and journaling, that promote self-connection. By understanding self-connection and finding ways to be more connected to oneself, we hope to help everyone pursue a life “well-lived.”
